# Probiotic *Lactobacillus plantarum* Ln4 Showing Antimicrobial and Antibiofilm Effect against *Streptococcus mutans* KCTC 5124 Causing Dental Caries

**DOI:** 10.4014/jmb.2306.06001

**Published:** 2023-09-08

**Authors:** Hye Ji Jang, Jong Ha Kim, Na-Kyoung Lee, Hyun-Dong Paik

**Affiliations:** Department of Food Science and Biotechnology of Animal Resources, Konkuk University, Seoul 05029, Republic of Korea

**Keywords:** Probiotics, *Streptococcus mutans*, dental caries, antimicrobial effect, antibiofilm effect

## Abstract

Dental caries has known as an infectious disease that is considered a serious global public health problem. Recently, report indicate that probiotics play a vital role in maintaining oral health. Therefore, this study aimed to evaluate the prevention effects of *Lactobacillus plantarum* Ln4 against dental infection by the pathogenic bacterium *Streptococcus mutans* KCTC 5124 through biofilm formation inhibition. To evaluate such prevention effects against *S. mutans* KCTC 5124, antimicrobial activity, auto-aggregation, co-aggregation, cell surface hydrophobicity, total exopolysaccharide (EPS) production rate, and biofilm formation were analyzed. Results showed that *L. plantarum* Ln4 showed higher antimicrobial activity than *L. rhamnosus* GG (LGG). In the group treated with *L. plantarum* Ln4, the co-aggregation (58.85%), cell surface hydrophobicity (16.75%), and EPS production rate (73.29%) values were lower than those of LGG and the negative control. Additionally, crystal violet staining and confocal laser scanning microscopy (CLSM) revealed that *L. plantarum* Ln4 effectively inhibited biofilm formation in *S. mutans* KCTC 5124. Therefore, *L. plantarum* Ln4 could be used in the industry as a probiotics to prevent and improve oral health.

## Introduction

Dental caries is one of the most common oral diseases worldwide and has been associated with various disorders [1, 2]. Environmental factors, including diet, oral bacteria, oral hygiene, and oral quality of life, contribute to the development of oral diseases including dental caries and periodontal diseases, leading to plaque biofilm formation on the tooth surface [1, 3].

Among several distinct oral pathogenic bacteria, *Streptococcus mutans*, an anaerobic, gram-positive bacteria, is a major contributor to dental caries [1, 2, 4]. One study indicated that *S. mutans* secretes glycosyltransferases (GTFs), which play a critical role in biofilm formation on the tooth [5]. Dental biofilms are related to microbial colonization on tooth surfaces, oral diseases, and dental caries. If dental caries are not eliminated, preexisting or new bacteria invade the tooth surface, leading to the maturation of the biofilm [6]. Although diverse antimicrobial compounds, including chlorhexidine, vancomycin, and ampicillin, have been effective in the treatment of dental caries, they could result in unexpected side effects such as the development of multidrug-resistant bacterial strains [7]. Notably, recent studies have indicated that probiotics play an important role in oral health [8].

Probiotics are nonpathogenic live microorganisms that provide health benefits to their host when administered in adequate amounts [9-12]. Probiotics have been shown to exert a wide range of biological functions such as modulation of the immune system, and show anti-inflammatory, antioxidant, antidiabetic, antiallergy, and anticancer effects that improve the health of the host [13-15]. Several studies have investigated the diverse characteristics of probiotics to develop products beneficial to human health. Characteristically, probiotics have certain properties that prevent the invasion and adhesion of pathogenic bacteria. Probiotics have gradually increased in popularity as they are now considered useful for preventing oral infections [16]. A recent study reported that *Lactobacillus* sp. can be used as food additives due to its diverse probiotic properties, including the secretion of organic acids, bacteriocins, and hydrogen peroxide [9, 17, 18]. In this respect, it has been suggested that probiotics, including *Lactobacillus* sp. and other strains, might have dental caries-reducing effects [19].

In a previous study, *Lactobacillus plantarum* Ln4 was found to exhibit various probiotic properties including high resistance to artificial gastric conditions, enzyme production, strong adhesion to HT-29 cells, and antioxidant and β-galactosidase activities [20]. The present study aimed to evaluate the antimicrobial and anti-biofilm potential of *L. plantarum* Ln4 against the oral pathogenic bacterium *Streptococcus mutans* KCTC 5124, assessing its efficacy in preventing dental caries.

## Materials and Methods

### Microorganisms and Culture Conditions

As oral pathogenic and probiotic strains, *S. mutans* and *Lactobacillus* strains were used for in vitro experiments. *L. plantarum* Ln4 and *L. plantarum* NK181 were isolated from kimchi and grown in lactobacilli MRS broth (BD Biosciences, USA). *Lactobacillus rhamnosus* GG (LGG), a commercial strain; obtained from the Korean Collection for Type Cultures (Republic of Korea), was used as the control strain. All *Lactobacillus* strains were grown on MRS broth at 37°C for 24 h.

*S. mutans* KCTC 5124, an oral pathogenic bacterium, was obtained from the Korean Collection for Type Culture. *S. mutans* KCTC 5124 was incubated in BHI broth (BD Biosciences) with 3% sucrose at 37°C for 24 h in a 5% CO_2_ incubator.

### Antimicrobial Activity of *Lactobacillus* Strains

The antimicrobial activity of *Lactobacillus* strains against *S. mutans* KCTC 5124 was assessed using a modified deferred method [21]. Briefly, 3 μl of *L. plantarum* strains (approximately 10^9^ CFU/ml) were spotted onto MRS agar and grown at 37°C for 24 h. Following incubation, 100 μl of *S. mutans* KCTC 5124 as an indicator oral pathogenic bacterium (approximately 10^6^ CFU/ml) was inoculated into 4 ml of BHI soft agar with 3% sucrose and overlaid. The overlaid plate was incubated at 37°C for 48 h in a 5% CO_2_ incubator. The diameter (mm) of the clear zones were measured. LGG spotted plates were used as controls.

### Minimum Inhibitory Concentration (MIC) of *Lactobacillus* Strains against *S. mutans* KCTC 5124

The MIC was determined using a previously described method with minor modifications [22]. In brief, *S. mutans* KCTC 5124 was seeded on a 96-well plate (2 × 10^6^ CFU/ml) and incubated at 37°C for 24 h in a 5% CO_2_ incubator. *Lactobacillus* strain cultures were centrifuged at 14,240 ×*g* for 5 min, and the supernatants were diluted two-fold in BHI broth. Diluted samples (50 μl) were added to the 96-well plate, except in the wells used as negative controls. The plate was then incubated at 37°C for 24 h in a 5% CO_2_ incubator. The MIC was defined as the lowest *L. plantarum* supernatant concentration that inhibited growth (no visible growth). Experiments were evaluated in triplicate at three different times.

### Cell Surface Properties

The auto-aggregation and coaggregation of *S. mutans* KCTC 5124 and *Lactobacillus* strains were determined by using some modifications [21]. Briefly, *Lactobacillus* and *S. mutans* strains were centrifuged at 14,240 ×*g* for 5 min and washed twice with PBS buffer. Cells were adjusted to an OD_600_ of 0.5 ± 0.05 with PBS buffer. Each bacterial suspension (4 ml) was incubated at 37°C for 4 h and 24 h to identify autoaggregation. The absorbance was measured at 600 nm after 4 and 24 h of incubation. Autoaggregation was calculated as follows:

Autoaggregation (%) = (1 – (OD_Time_ / OD_Initial_)) × 100

To determine co-aggregation, *Lactobacillus* strains suspension and an *S. mutans* KCTC 5124 suspension were mixed and incubated at 37°C for 4 h and 24 h. Absorbance was measured at 600 nm and co-aggregation was calculated as follows:

Coaggregation (%) = (1 – (OD_Mix_ / (OD_P_ + OD_L_) / 2)) × 100

where OD_p_, OD_L_, and OD_Mix_ represent the absorbances of the cultures of pathogenic bacterium, *Lactobacillus* strains, and mixed strains, respectively.

### Hydrophobicity Determination

The hydrophobicity of *S. mutans* KCTC 5124 treated *Lactobacillus* strains was determined as previously described, with some modifications [22, 24]. Xylene was used to characterize hydrophobicity. Briefly, culture strains were incubated in MRS broth at 37°C for 24 h and centrifuged at 14,240 ×*g* for 5 min; culture supernatants were washed twice and resuspended in PBS buffer. Next, resuspended cells were adjusted to an OD_600_ of 0.5 (OD_Initial_). Resuspended cells (3 ml) were then added to 1 ml of solvent (xylene), pre-incubated at 37°C for 10 min in an incubator, and incubated at 37°C for 20 min. After incubation, the mixture was separated into two phases. The aqueous phase (1 ml) was collected, and the its absorbance was measured at 600 nm (OD_Time_). Hydrophobicity was calculated as follows:

Cell surface hydrophobicity (%) = (1 – (OD_Time_ / OD_Initial_)) × 100

### Total Exopolysaccharide (EPS) Production Rate

The total EPS production rate was evaluated as previously described, with some modifications [23]. Briefly, *Lactobacillus* strains and *S. mutans* KCTC 5124 were grown in MRS broth and BHI broth with 3% sucrose at 37°C for 24 h in a 5% CO_2_ incubator. *S. mutans* KCTC 5124 was mixed with the MIC of *Lactobacillus* and LGG strains in BHI broth containing 3% sucrose at 37°C for 24 h in an anaerobic incubator. The suspensions were centrifuged at 8,000 ×*g* for 10 min, and the supernatants were collected. Supernatant samples (1 ml) were added to 2 ml of 99%ethyl alcohol and incubated at 4°C for 24 h. The mixtures were centrifuged at 14,240 ×*g* for 15 min, and the pellets were resuspended by using distilled water (500 μl). Cell suspensions (40 μl) were combined with 40 μl of 5%phenol and 4 ml of 95% sulfuric acid, and the reaction was carried out for 10 min at 30°C. Absorbance was measured to determine the total EPS production rate, which was calculated as follows:

EPS production rate (%) = (OD_Treatment_ / OD_Control_) × 100

### Biofilm Formation Using Crystal Violet Staining

Biofilm formation was evaluated using the method reported by Lim *et al*. [8] with some modifications. Briefly, *S. mutans* KCTC 5124 was seeded on a 24-well plate (1 × 10^6^ CFU/ml) and incubated at 37°C for 48 h in an anaerobic incubator. Each well was then inoculated with the supernatant of *Lactobacillus* strains, or LGG at a concentration of 10^9^ CFU/ml (100 μl, 200 μl, and 500 μl per well) and incubated at 37°C for 15 h in a 5% CO_2_ incubator. After incubation is over, the planktonic mixtures were removed and washed three times with PBS buffer. The plates were dried at 37°C for 10 min, stained using 0.1% crystal violet solution for 10 min. Next, the stained plates were washed, rinsed with distilled water, and air-dried completely at room temperature. A solvent mixture (10% acetic acid, 30% methanol, and 60% distilled water) was added to each well and shaken until crystal violet dissolved. Absorbance was measured at 570 nm, and the biofilm inhibition rate was calculated as follows:

Biofilm inhibition rate (%) = (1 – (OD_Sample_ / OD_Control_)) × 100

### Confocal Laser Scanning Microscopy (CLSM)

CLSM was conducted to quantitatively evaluate the inhibition of *S. mutans* KCTC 5124 biofilm formation by *Lactobacillus* strains, with modifications [23]. Briefly, *S. mutans* KCTC 5124 was cultured in BHI broth containing 3% sucrose at 37°C for 24 h in a 5% CO_2_ incubator, and *Lactobacillus* and LGG strains were cultured in MRS broth at 37°C for 24 h. *S. mutans* KCTC 5124 was seeded in a 6-well plate (1 × 10^6^ CFU/ml), and glass coupons were added. *Lactobacillus* and LGG strains were treated with MIC concentration, while the control group was treated with BHI broth containing 3% sucrose. After incubation at 37°C for 24 h, glass coupons were washed with PBS buffer and stained with 1 μM/ml of SYTO9 (Invitrogen, Thermo Fisher Scientific, USA) for 20 min in the dark at room temperature. Glass coupons were then washed twice with PBS buffer and air-dried for 40 min in the dark. Glass coupons were fixed with coverslips and observed using a confocal laser scanning microscopy (Carl Zeiss, Germany).

### Statistical Analysis

Results for each treatment were obtained in triplicate, and one-way analysis of variance (SPSS software version 19; IBM, USA) and Student’s *t*-test were performed to determine the significance of the differences among the mean values. Results are presented as the mean ± standard deviation.

## Results

### Antimicrobial Effect against *S. mutans* KCTC 5124

The antimicrobial activity of *Lactobacillus* strains against *S. mutans* is shown in Table 1. In general, *L. plantarum* strains exhibited better antimicrobial activity than LGG. Particularly, *L. plantarum* Ln4 showed a large clear zone (30.33 mm) against oral pathogenic *S. mutans* KCTC 5124 (*p* < 0.05). Although LGG showed the largest clear zone (31.33 mm), no statistically significant differences were found between the clear zones formed by *L. plantarum* Ln4 and LGG.

We determined the MICs of *Lactobacillus* strains and LGG against the oral pathogenic bacterium *S. mutans* KCTC 5124. *L. plantarum* Ln4, *L. plantarum* NK181, and LGG inhibited *S. mutans* KCTC 5124 growth at concentrations of 12.5%, 6.25%, and 12.5%, respectively. LGG was used as the positive control strain compared with *Lactobacillus* strains (data not shown).

### Cell Aggregation and Cell Surface Hydrophobicity

The effects of *Lactobacillus* strains on auto-aggregation, co-aggregation, and cell surface hydrophobicity of *S. mutans* KCTC 5124 were shown in Table 2. After 4 h of incubation, *S. mutans* KCTC 5124 showed a low autoaggregation value (18.23%); however, it increased after 24 h of incubation (70.99%).

Coaggregation activity of *Lactobacillus* strains and LGG with *S. mutans* KCTC 5124 was evaluated during incubation (4 h and 24 h). *L. plantarum* Ln4, *L. plantarum* NK181, and LGG showed 17.37%, 16.42%, and 18.24%coaggregation, respectively, at 4 h. Although no difference was observed at 4 h, these strains showed considerable difference after 24 h. Coaggregation values of *L. plantarum* Ln4, *L. plantarum* NK181, and LGG were recorded as 58.85%, 54.21%, and 53.66%, respectively, at 24 h.

**Fig. 1:**
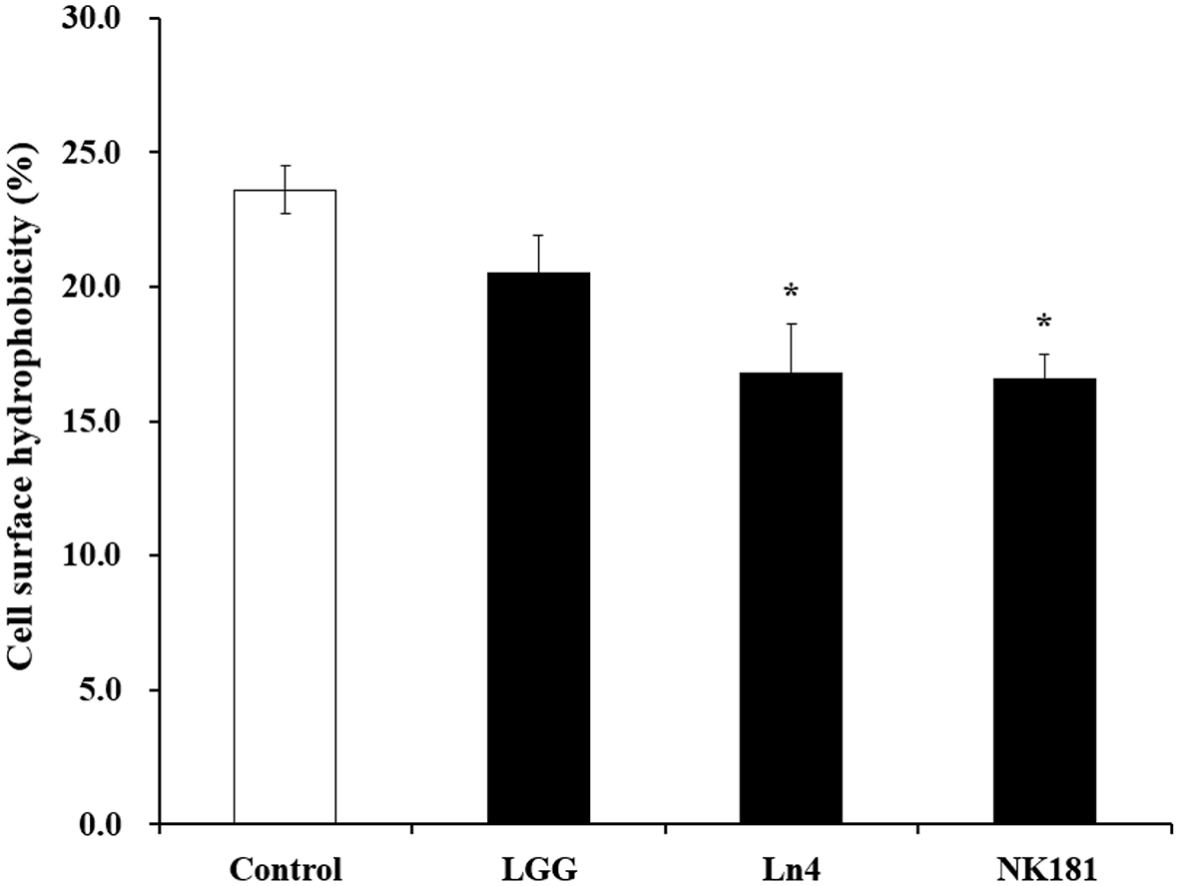
Changes in cell surface hydrophobicity of oral pathogenic bacterium *Streptococcus mutans* KCTC 5124 treated with *Lactobacillus* strains. □, Control (untreated with *Lactobacillus* strains); ■, treated with LAB. LGG, *L. rhamnosus* GG (12.5% treatment); Ln4, *L. plantarum* Ln4 (12.5% treatment); NK181, *L. plantarum* NK181 (6.25% treatment). Each value represents the mean ± standard deviation, and different letters on each bar represent a significant difference between values (*p < 0.05).

In addition, the cell surface hydrophobicity was measured by bacterial adhesion to hydrocarbons, when compared to the control and treated *Lactobacillus* strains [25, 26]. The cell surface hydrophobicity of *L. plantarum* strains against *S. mutans* KCTC 5124 was associated with its adhesion ability, as shown in Fig. 1. The control group (*S. mutans* KCTC 5124), which is untreated with *Lactobacillus* strains, showed 23.60% cell surface hydrophobicity. However, *L. plantarum* Ln4 (16.75%) and *L. plantarum* NK181 (16.56%) treated groups had significantly reduced cell surface hydrophobicity values (*p* < 0.05).

### Total EPS Production Rate

**Fig. 2:**
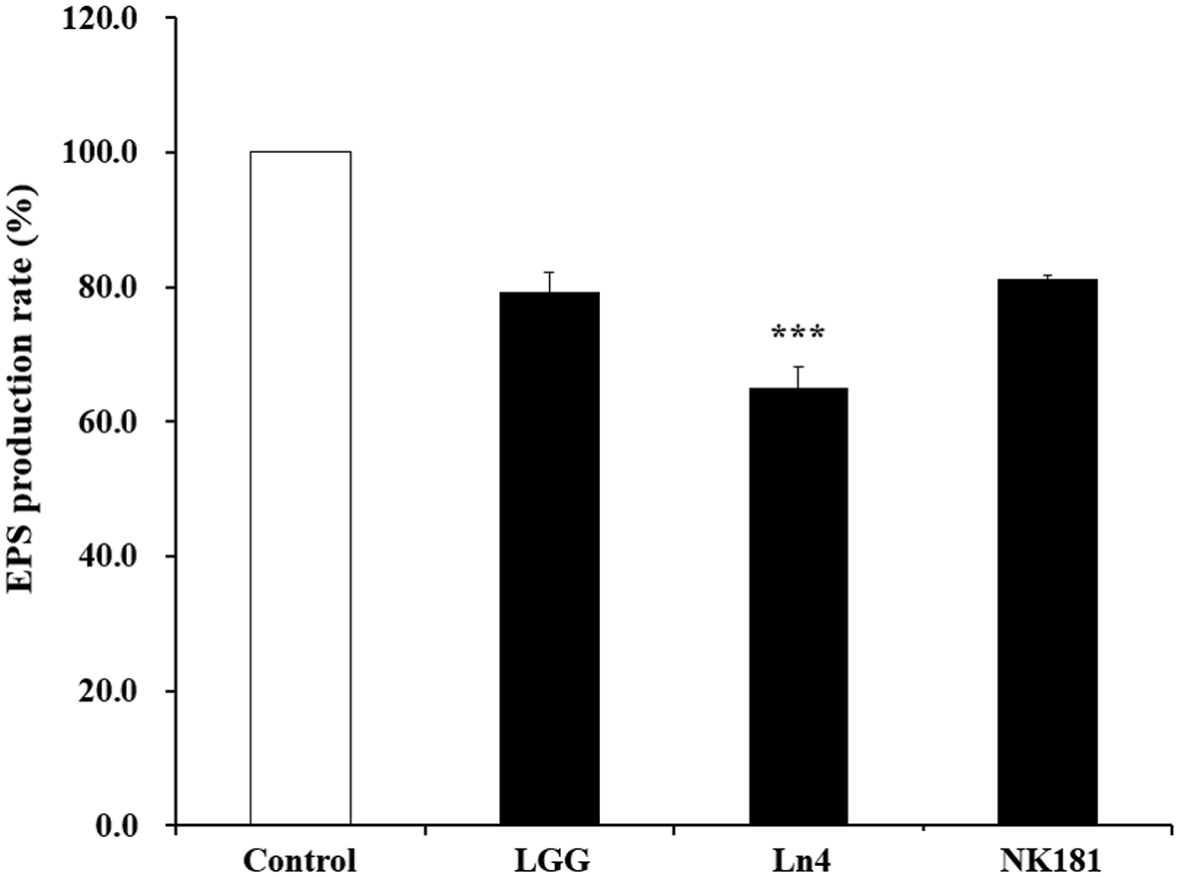
EPS production rate of oral pathogenic bacterium *Streptococcus mutans* KCTC 5124 treated with *Lactobacillus* strains. □, Control (untreated with *Lactobacillus* strains); ■, treated with LAB. LGG, *L. rhamnosus* GG (12.5% treatment); Ln4, *L. plantarum* Ln4 (12.5% treatment); NK181, *L. plantarum* NK181 (6.25% treatment). Each value represents the mean ± standard deviation, and different letters on each bar represent a significant difference between values (***p < 0.001).

When the total EPS production rate of *S. mutans* KCTC 5124 was studied, the results indicated that *Lactobacillus* strains led to a reduction in the total EPS production by *S. mutans* KCTC 5124 (Fig. 2). Among the *Lactobacillus* strains, *L. plantarum* Ln4 significantly reduced the total EPS production (34.98%), and following, *L. plantarum* NK181 also showed an inhibitory effect on EPS production (18.85%). These results were significantly different from those of the control (untreated *Lactobacillus* stains) (*p* < 0.001).

### Biofilm Formation and CLSM

**Fig. 3:**
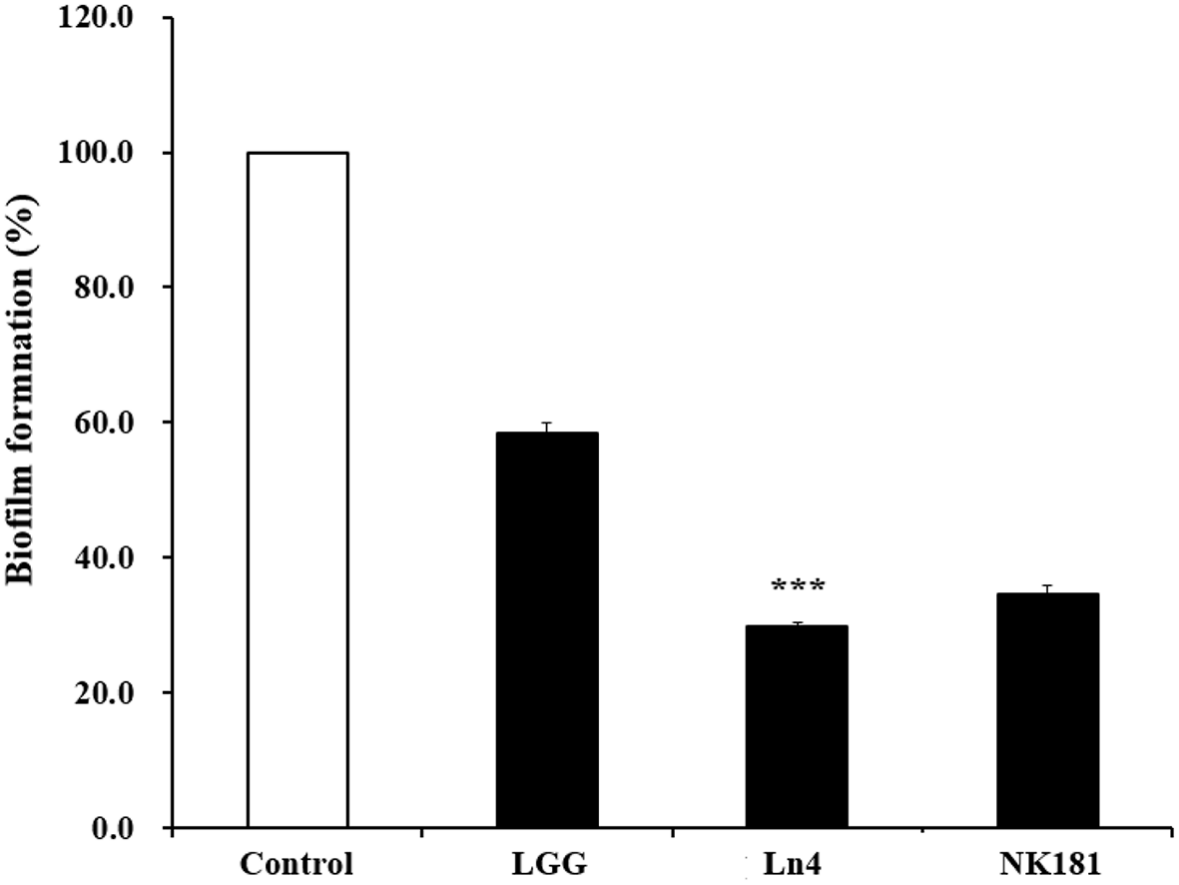
Effect of *Lactobacillus* strains on the biofilm formation of oral pathogenic bacterium *Streptococcus mutans* KCTC 5124. □, Control (untreated with *Lactobacillus* strains); ■, treated with LAB. LGG, *L. rhamnosus* GG (12.5% treatment); Ln4, *L. plantarum* Ln4 (12.5% treatment); NK181, *L. plantarum* NK181 (6.25% treatment). Each value represents the mean ± standard deviation, and different letters on each bar represent a significant difference between values (***p < 0.001).

Lactobacillus strains inhibited biofilm formation by *S. mutans* KCTC 5124 (Fig. 3). *L. plantarum* Ln4 showed the highest inhibitory effect on *S. mutans* KCTC 5124 biofilm formation, reducing it to 34.64% compared to that of the control (*p* < 0.001) (Fig. 3). *L. plantarum* NK181 and LGG also inhibited biofilm formation by *S. mutans* KCTC 5124.

**Fig. 4:**
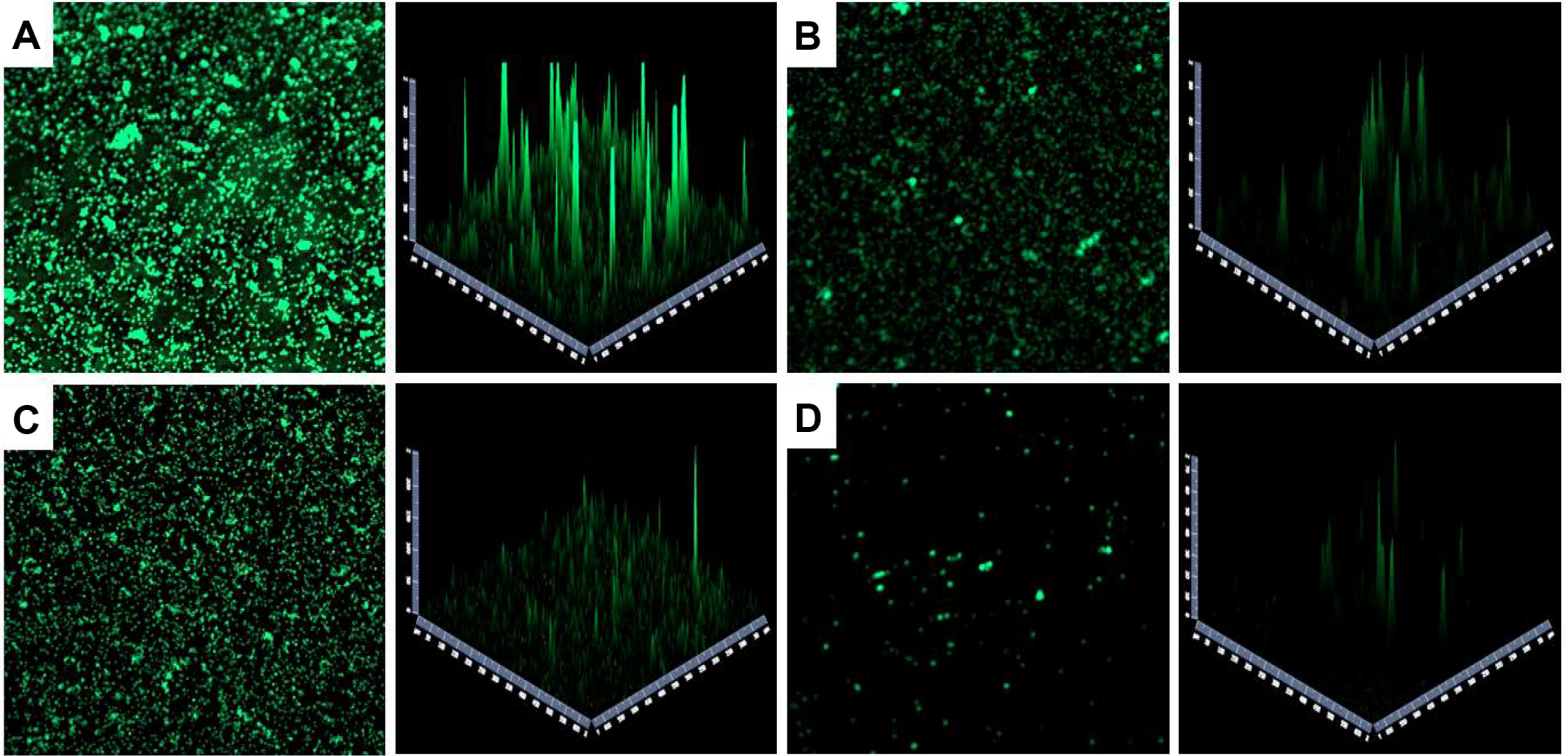
Inhibition of biofilm by *S. mutans* KCTC treated with cell-free supernatant (CFS) of *L. plantarum* Ln4 visualized by confocal laser scanning microscopy (CLSM) (× 50 magnification). (**A**) Control group (0% treatment); (**B**) treated with *L. rhamnosus* GG (12.5% treatment); (**C**) treated with *L. plantarum* NK181 (6.25% treatment); (**D**) treated with *L. plantarum* Ln4 (12.5% treatment).

CLSM was used to evaluate biofilm formation inhibition by *L. plantarum* Ln4 (Fig. 4). *S. mutans* KCTC 5124 and LGG were used as negative and positive controls, respectively. Compared to that of negative control, biofilm formation was reduced in *Lactobacillus*-strains and LGG-were treated groups. Among them, *S. mutans* KCTC 5124 treated with *L. plantarum* Ln4 showed the highest biofilm inhibition.

## Discussion

Dental caries is known as a major disease related with oral condition, which is multi-species biofilm-mediated [16]. It has been previously reported that probiotics promote oral health. Specifically, *S. mutans* is a major oral pathogenic bacteria associated with dental caries, and antimicrobial activity plays a vital role suppressing these dental caries [27]. The biofilm formed by *S. mutans* secretes glucosyltransferases that synthesize glucans to promote bacterial binding (adhesion) to the tooth surface. Adhesion is thus critical for biofilm progression [28]. Biofilms are formed by microbial communities to resist a variety of conditions and to protect bacterial cells by attaching tenaciously to each other [5, 29]. Consequently, control of early step is important to inhibit biofilm-formation by *S. mutans* [5]. The biofilm can be suppressed by antimicrobial activity, which could be affected by organic acids, hydrogen peroxide, bacteriocin, and biosurfactants [7, 30]. This study evaluated the antimicrobial effect of *L. plantarum* stains against *S. mutans* KCTC 5124. Our findings showed that *L. plantarum* antimicrobial and antibiofilm activities inhibited biofilm formation by *S. mutans* KCTC 5124. Among the tested *Lactobacillus* strains, *L. plantarum* Ln4 showed the highest antimicrobial activity against *S. mutans* KCTC 5124 using the deferred method and MIC test. In particular, compared to *L. plantarum* 200661, *L. plantarum* Ln4 showed higher antibacterial activity at the same concentration [7]. Consequently, we evaluated that *L. plantarum* Ln 4 could use as potential strain when compared to *Weissella cibaria* CMU and *Lactobacillus reuteri* DSM 17938 (widely recognized oral probiotics) [31, 32].

Generally, autoaggregation, cell surface hydrophobicity, and EPS production are related to bacterial adhesion to the tooth surface and are important elements to consider when aiming to prevent biofilm formation by *S. mutans* [16]. It has been proven that bacteria can better colonize the tooth surface when they exhibit high hydrophobic activity. Especially, biofilm formed by *S. mutans* related with sucrose and hydrophobic activity results in attached to tooth surface [26].

In addition, EPS are the major factor in forming, maturing, maintaining, and expending the *S. mutans* biofilm matrix. Thus, we investigated the effects of autoaggregation, hydrophobicity, and EPS production on *L. plantarum* Ln4 against *S. mutans*. We determined that *L. plantarum* Ln4 had the greatest effects of aggregation, hydrophobic activity, and EPS production changes among the tested *Lactobacillus* strains. Moreover, regarding cell surface properties, *L. plantarum* Ln4 significantly reduced EPS production by *S. mutans* (*p* < 0.001). Taken together, results suggest that *L. plantarum* Ln4 might be expected to prevent cavity by inhibiting the aggregation of *S. mutans*.

A previous study reported that coaggregation of *Lactobacillus* sp. strains with *S. mutans* ATCC 25175 varied between 6.32% to 20.93%. In addition, compared to the autoaggregation of *S. mutans* ATCC 25175, the coaggregation of *S. mutans* ATCC 25175 with *L. plantarum* sp. strains was low [31]. Another study demonstrated that *L. plantarum* K25 decreased EPS formation (21.44%) as well as the antimicrobial peptide GH12, which has a dental caries effect at 1/4 and 1/2 MIC, remarkably reduced EPS [33].

We also conduct to CLSM analysis to measure the bacterial counts as staining cells in biofilm [34]. CLSM as microscopy methods confirmed inhibition of biofilm by *S. mutans* in treated *Lactobacillus* strains. Among the strains, *L. plantarum* Ln4 showed the highest biofilm formation inhibition (Fig. 4). In general, it is difficult to remove a mature biofilm than early biofilm and then biofilm degradation is vital to measure the antibiofilm activity against *S. mutans*. Because biofilm protect oral bacteria and *S. mutans* return cellular damage by stress through induce membrane protein [16]. One study reported that *L. plantarum* FB-T9 inhibited biofilm formation by *S. mutans* depending on the incubation time, when compared to the control [35]. In addition, another study demonstrated that *Lactobacillus* strains inhibit biofilm formation by *S. mutans* [19]. *L. brevis* KCCM 202399 reported that the highest antibiofilm effect against *S. mutans* KCTC 5458 at MIC levels [16]. It has been reported that coaggregation with lactic acid bacteria and reduces EPS production by physical interference.

This study demonstrated that *L. plantarum* Ln4 has potential effects to prevent dental caries using its antimicrobial activity and to be applied as food additives in oral health industry.

## Conclusion

The oral health effects of probiotics have recently been investigated. Among the oral pathogenic bacteria, *S. mutans* is major bacteria that influences dental caries. *L. plantarum* Ln4 was previously evaluated for probiotic characteristics and other functional activities. In this study, our findings demonstrated the antimicrobial and antibiofilm activities of *L. plantarum* Ln4 against the oral pathogenic bacterium *S. mutans* KCTC 5124 through autoaggregation, coaggregation, cell surface hydrophobicity, EPS production rate, and inhibition of biofilm formation analyses. *L. plantarum* Ln4 was effective when compared with the control, which was not treated with *Lactobacillus* strains, in all experiments. Therefore, *L. plantarum* Ln4 could inhibit biofilm formation of oral pathogenic bacteria and is expected to be used in the healthcare industry.

## Figures and Tables

**Table 1:** Inhibition activity of *Lactobacillus* strains against oral pathogenic bacterium *Streptococcus mutans* KCTC 5124.

Pathogenic bacterium	Inhibitory diameter (mm)		
	LGG^1)^	*L. plantarum* NK181	*L. plantarum* Ln4
*S. mutans* KCTC 5124	18.63 ± 0.40	31.33 ± 5.86*	30.33 ± 6.66*

^1)^LGG, *L. rhamnosus* GG

All values are mean ± standard deviation (*p < 0.05).

**Table 2:** Autoaggregation and coaggregation of *Lactobacillus* strains against oral pathogenic bacterium *Streptococcus mutans* KCTC 5124.

Microorganisms	Time (h)	
	4 h	24 h
Auto-aggregation (%)		
*S. mutans* KCTC 5124	18.23 ± 1.14^Ba^	70.99 ±2.48^Aa^
Co-aggregation (%)		
LGG^1^	18.24 ± 0.93^Ba^	53.66 ± 2.61^Ac^
*L. plantarum* Ln4	17.37 ± 0.62^Ba^	58.85 ± 2.27^Ab^
*L. plantarum* NK181	16.42 ± 1.41^Bb^	54.21 ± 1.44^Ac^

^1)^LGG, *L. rhamnosus* GG

^A-B^ The superscript uppercase letters in the same row indicate statistical differences by Student’s *t*-test (*p* < 0.05)

^a-c^The superscript lowercase letters in the same column indicate statistical differences by ANOVA (*p* < 0.05)
